# My Body Looks Like That Girl’s: Body Mass Index Modulates Brain Activity during Body Image Self-Reflection among Young Women

**DOI:** 10.1371/journal.pone.0164450

**Published:** 2016-10-20

**Authors:** Xiao Gao, Xiao Deng, Xin Wen, Ying She, Petra Corianne Vinke, Hong Chen

**Affiliations:** 1 Key Laboratory of Cognition and Personality, Southwest University, Chongqing, China; 2 Faculty of Psychology, Southwest University, Chongqing, China; 3 Stomatological Hospital of Chongqing Medical University, Chongqing, China; 4 Chongqing Key Laboratory of Oral Diseases and Biomedical Sciences, Chongqing, China; 5 Chongqing Municipal Key Laboratory of Oral Biomedical Engineering of Higher Education, Chongqing, China; 6 Groningen Institute of Evolutionary Life Sciences, Dept. Behavioral Neurosciences, University of Groningen, Groningen, The Netherlands; Institute of Psychology, Chinese Academy of Sciences, CHINA

## Abstract

Body image distress or body dissatisfaction is one of the most common consequences of obesity and overweight. We investigated the neural bases of body image processing in overweight and average weight young women to understand whether brain regions that were previously found to be involved in processing self-reflective, perspective and affective components of body image would show different activation between two groups. Thirteen overweight (O-W group, age = 20.31±1.70 years) and thirteen average weight (A-W group, age = 20.15±1.62 years) young women underwent functional magnetic resonance imaging while performing a body image self-reflection task. Among both groups, whole-brain analysis revealed activations of a brain network related to perceptive and affective components of body image processing. ROI analysis showed a main effect of group in ACC as well as a group by condition interaction within bilateral EBA, bilateral FBA, right IPL, bilateral DLPFC, left amygdala and left MPFC. For the A-W group, simple effect analysis revealed stronger activations in Thin-Control compared to Fat-Control condition within regions related to perceptive (including bilateral EBA, bilateral FBA, right IPL) and affective components of body image processing (including bilateral DLPFC, left amygdala), as well as self-reference (left MPFC). The O-W group only showed stronger activations in Fat-Control than in Thin-Control condition within regions related to the perceptive component of body image processing (including left EBA and left FBA). Path analysis showed that in the Fat-Thin contrast, body dissatisfaction completely mediated the group difference in brain response in left amygdala across the whole sample. Our data are the first to demonstrate differences in brain response to body pictures between average weight and overweight young females involved in a body image self-reflection task. These results provide insights for understanding the vulnerability to body image distress among overweight or obese young females.

## Introduction

Obesity is becoming a worldwide problem as it is a complex condition with serious social and psychological dimensions. It affects nearly all age and all socioeconomic groups and threatens to overwhelm both developed and developing countries (WHO, http://www.who.int/nutrition/topics/obesity/en/). Body dissatisfaction or body image distress is one of the most common consequences produced by obesity and overweight [1]. In previous studies, body mass index (BMI) was found not only to be a strong predictor of body dissatisfaction [2, 3], but also to have long term negative effects on body image [4]. Therefore, elucidating body image processing among obese or overweight individuals would help to understand the social and psychological experience of being obese, and could then provide guidelines to improve health care for this population [5].

Modern western culture emphasizes extreme and unrealistic thinness, denigrates excess weight, and stigmatizes obese or overweight individuals. These cultural factors play a vigorous role in shaping body image among the overweight or obese individuals [5–7]. Appearance related social comparison bridges the sociocultural factors and negative body image. Although a large amount of research has focused on the impact of appearance-based social comparison, little is known about its brain mechanisms. One important aspect underlying appearance-based social comparison is body image representation. Functional neuroimaging studies in healthy women and patients with eating disorders reported that body image representation in the human brain has at least two main networks [8–16]. The first network represents perceptive processing of body image and can be activated when viewing subjects’ own body, others’ body or body line drawings. This brain network consists of the inferior parietal lobule (IPL), extrastriate body area (EBA, lateral occipitotemporal cortex) and fusiform body area (FBA) [8, 10, 12, 17–20]. The second network is related to affective processing of body image and can be activated when viewing body pictures with negative emotional valence. For example, when viewing self-distorted fat body photos, dorsolateral prefrontal cortex (DLPFC) and amygdala are usually activated. These findings were reported among both young healthy women and anorexia nervosa (AN) patients [13, 14, 21, 22]. This network comprises of prefrontal cortex–limbic/paralimbic structures [13, 14, 21, 22].

Another important aspect underlying appearance-based social comparison is self-reflection. When body-shape stimuli or tasks carry significant self-referent information, medial prefrontal cortex (MPFC) was reported to be activated [9, 10, 21, 23]. MPFC has consistently been found to be implicated in self-reflection tasks [24–26]. However, research on neural correlates of self-reflective body image processing appears underdeveloped relative to the theoretical and empirical importance of body image in many current psychological models of self-evaluation or eating disorders [27–31]. There have been only two studies, to the authors’ knowledge, that have directly looked at this. One study among normal weight healthy women showed a stronger activation of bilateral MPFC during fat-body self-reflection relative to thin-body self-reflection [9]. The second study reported greater MPFC activation in bulimia nervosa patients than in healthy control participants during fat-body self-reflection [32].

A third factor that may modulate neural response of body image processing is body dissatisfaction. Extreme examples come from studies in AN patients, who show an overestimation of own body size, a greater body dissatisfaction and greater self-ideal discrepancies [13, 21, 23, 33]. Functional MRI studies in AN patients that primarily focus on the affective component of the body image processing reported altered activation of PFC and hyperactivation of amygdala, ACC, and insula [34]. Findings from studies among healthy women have also shown that PFC, ACC and amygdala are implicated in body dissatisfaction induced by exposure to self-distorted fat images [10, 13, 21, 22] or attractive slim bodies of another woman [8].

However, there have been little neuroimaging studies investigating body image processing among overweight or obese individuals. This is unfortunate in light of the empirical evidence, which showed that overweight and obesity is linked to poor body image and higher body image distress, which could in turn lead to additional psychological distress and binge eating (reviewed in [5, 35–37]). Therefore, by using a body image self-reflection paradigm, the present study sought to first investigate whether brain regions previously found to be involved in body image processing would show different activation in overweight compared to average weight young women. If such group differences would be found, it was aimed to determine whether these group differences in neural response to body image processing were mediated by bodyweight dissatisfaction. The virtual body image stimuli and body image self-reflective task used in this study have also been used in previous *f*MRI studies [9, 32]. The task was shown to be able to elicit self-body representation/interoception, and could activate the MPFC, which was most implicated in self-referential and self-evaluative thought. We hypothesized that in this body image self-reflection task: (1) compared to the average weight group, overweight group would have a stronger activation to fat or thin body images vs. control images in brain regions related to affective components of body image processing; (2) body dissatisfaction would partially or completely mediate the group differences in neural response to fat or thin body images vs. control images within these affective-related regions; and (3) compared to the average weight group, overweight group would show an altered activation to fat or thin body images vs. control images in brain regions relating to body image self-reflection (MPFC).

## Materials and Methods

### Participants

Fifteen average weight women and 16 overweight women enrolled at Southwest University, China, participated in the current study. However, three overweight and two average weight participants were excluded due to excessive head movement (> 1 mm at any of the six directions) during the *f*MRI image acquisition. The final sample consisted of 13 women in the average weight group (A-W) and 13 women in the overweight group (O-W). All participants were right-handed non-smokers, with no reported past/current neurological or psychiatric illness, normal or corrected-to-normal vision and normal color vision as assessed by basic color tests. Demographic information and self-reported data on bodyweight dissatisfaction, eating behaviors and appearance-related social comparison tendency were measured. The results are presented in Table 1.

**Table 1:** Demographic Information and self-reported data of Participants: Mean (Standard Deviation).

	O-W Group	A-W Group	*F*	*p*
*n*	13	13		
Age (years)	20.31 (1.70)	20.15 (1.62)	0.06	0.80
BMI (kg/m^2^)	25.33 (1.41)	19.07 (1.48)	35.84	< .001
NPS-F	2.19 (0.40)	1.43 (0.41)	771.10	< .001
DEBQ				
Restrained eating	31.61 (6.32)	25.62 (4.93)	7.29	0.01
Emotional eating	33.61 (10.65)	27.62 (6.79)	2.93	0.10
External eating	35.16 (7.83)	31.08 (6.06)	2.20	0.15
PACS	2.17 (1.00)	1.54 (0.71)	3.66	0.07
STAI-T	2.11 (0.41)	2.26 (0.65)	0.45	0.51

Note: O-W Group = Overweight group; A-W Group = Average weight group; NPS-F = Negative Physical Self Scale-Fatness; DEBQ = Dutch Eating Behavior Questionnaire; PACS = Physical Appearance Comparison Scale; STAI-T = The State-Trait Anxiety Inventory-Trait subscale

### Measures

Demographics. Participants’ age and grade in university were assessed. BMI was calculated from objectively-measured height and weight [BMI = weight(kg)/height^2^(m^2^)].

Negative Physical Self Scale-Fatness subscale (NPS-F) [38]. The 11-item Fatness subscale of Negative Physical Self Scale measuring thoughts, feelings, and behaviors related to body weight dissatisfaction was used to assess body dissatisfaction. Each item was rated between *0 = not at all like me* and *4 = very much like me*. Sample items include “I am very distressed when I think about my weight,” “I am fat in others’ eyes,” and “I have tried many ways to lose weight.” Scores were obtained by summing the scores of all items and dividing this total by 11 to yield an average score ranging from 0 to 4. Higher scores reflect a higher level of dissatisfaction. The NPS-F yields internally consistent scores (*α* = .88), as well as stable scores over three weeks (*r* = .89) among female and male middle and high school students and undergraduates, and over nine months (*r* = .70) among middle school and high school girls [39]. It has satisfactory convergent and predictive validity [40] among samples of adolescents and young adults. Its alpha coefficient for the current study was *α* = .91.

The Dutch Eating Behavior Questionnaire (DEBQ) [41]. The DEBQ was used to measure eating behaviors. The questionnaire consists of 33 items covering three subscales that assess restrained, emotional, and external eating behavior. Response categories ranged from *1 = never* to *5 = very often*. This scale has good internal reliability and good concurrent, construct and predictive validity in clinical as well as non-clinical samples [42–44]. In this study, Cronbach’s alpha coefficients of the three subscales were .85, .88 and .80 for restrained, emotional and external eating, respectively.

The Physical Appearance Comparison Scale (PACS) [45]. PACS was used to measure social comparison on one’s appearance. The scale has four items which reflect the degree of overall appearance comparison to other individuals. Response options ranged from *1 = never* to *5 = always*. Scores were summed to form a total appearance comparison variable, with higher scores indicating higher level of comparison. Thompson et al. (1991) found that the PACS had adequate psychometric properties. In this sample, the PACS had an alpha of *α* = .86.

The State-Trait Anxiety Inventory-Trait subscale (STAI-T) [46]. STAI-T subscale is a brief self-rating scale assessing trait anxiety in adults. It consists of 20 items that evaluate how the respondent “generally” feels. The STAI-T asks participants to rate the frequency of their feelings on a 4-point Likert scale ranging from *1 = almost never* to *4 = almost always*. Sample items include “I am a steady person,” “I lack self-confidence,” and “I feel at ease”. Scores were obtained by summing the scores of all items and dividing this total by 20 to yield an average score ranging from 1 to 4—higher scores reflecting a higher level of trait anxiety. Its alpha coefficient for the current study was *α* = .91.

### Stimuli

Fat and Thin Female Body Images. Eighty-four images picturing 42 fat and 42 thin female bodies were used in the current study. All images were derived from Owens et al. (2010). Fifteen female undergraduate students who were not involved in the *f*MRI study rated all images on perceived body weight (from *1 = very slim* to *5 = very obese*), attractiveness (from *1 = very unattractive* to *5 = very attractive*), valence (from *1 = very negative* to *5 = very positive*) and arousal (from *1 = very peaceful* to 5 = very excited) on 5-point Likert scales. The fat and thin body image types were rated to differ in perceived body weight [*F* (1, 14) = 48.15, *p* < .001], attractiveness [*F* (1, 14) = 58.87, *p* < .001], valence [*F* (1, 14) = 35.70, *p* < .001] and arousal [*F* (1, 14) = 56.33, *p* < .001]. Fat body images were rated as more obese, less attractive, less valenced and less aroused than thin body images. In addition, subjects who participated in the *f*MRI study rated the similarity between their own body and the body of the models in the stimuli images on a 5-point Likert scale from *1 = very different* to *5 = very similar*. The control condition stimuli were derived from test stimuli using an image scrambling procedure.

### Procedure

Following approval from the Human Research Ethics Committee at SWU, participants were recruited via on-campus advertisements. Subsequently, 31 female undergraduate students engaged in the initial phase of the study. After reading a general overview of the study and signing an informed consent, participants completed individually-administered self-reported measures of age, handedness, history of neurological or psychiatric illness, NPS-F, DEBQ, PACS and STAI-T. Also, participants rated the similarity of their own body with the body of the models in the stimuli pictures. Objective weight and height measurements were taken and a vision test was completed. Last, participants were scheduled to take part in the scanning phase. Participants with BMI between 22.00–27.99 were assigned to the overweight group and those with BMI between 18.00–21.99 were assigned to the average weight group. Participants were asked to consume regular meals before the experiment. Scanning sessions took place between 9:00–11:00 am and 15:00–17:00 pm.

Functional MRI scanning. Visual images were presented in a block design. Using a Latin Square design, the experiment comprised one run with six blocks of the fat body condition, six blocks of the thin body condition and six blocks of the control condition. Each block consisted of seven images presented for 2 s each, resulting in a duration of 14 s per block. After each block, a white fixation cross was presented in the middle of the screen for 16 s. Based on this design, this run had a total duration of 560 seconds, including an additional 20 second rest period at the beginning of the scan run.

Participants were instructed to view each fat and thin body image closely, and vividly imagine that someone was comparing her body to the body in the picture. That is, imagine someone saying “your body looks like hers.” When the scrambled control stimuli were presented, participants were instructed simply to look at the images. After the scan, participants were debriefed about the research purposes and paid 50 Yuan as compensation for their time.

### Imaging data acquisition

Images were acquired in a 3T Siemens TRIO MRI scanner. Functional data comprised 280 volumes acquired with T2*-weighted gradient echo planar imaging (EPI) sequences. We obtained 32 echo planar images per volume sensitive to blood oxygenation level-dependent (BOLD) contrast (TR = 2000 ms; TE = 30 ms; 3 mm × 3 mm in-plane resolution; field of view [FOV] = 192). Slices were acquired in an inter-leaved order and oriented parallel to the AC–PC plane, with thickness of 3 mm, 0.99 mm gap. High-resolution T1-weighted 3D fast-field echo (FFE) sequences were obtained for anatomical reference (176 slices, TR = 1900 ms; TE = 2.52 ms; slice thickness = 1 mm; FOV = 250; voxel size = 1 mm × 1 mm × 1 mm).

### Imaging data analysis

Data were analyzed using the Brain Voyager QX v2.3 software (Brain Innovation, The Netherlands). Functional scans were realigned within and across runs to correct for head motion, and co-registered with each participant’s anatomical data. Functional data were then normalized into standard stereotactic Talairach space, resliced into a voxel size of 3mm× 3mm × 3mm and smoothed with a 6mm (full width half maximum) Gaussian kernel to increase signal-to-noise ratio. First level effects were estimated using the general linear model and employing a canonical hemodynamic response function convolved with the experimental design. Group analysis were conducted using random-effects models in order to enable population inferences.

**Fig 1:**
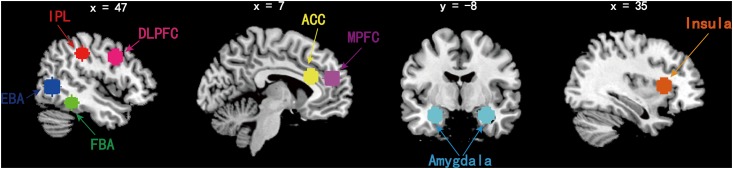
Sphere masks encompassing the IPL, EBA, FBA, ACC, MPFC, DLPFC, amygdala and insula used in ROI analyses of *f*MRI data. Red, inferior partial lobe (IPL); blue, extrastriate body area (EBA); green, fusiform body area (FBA); pink, dorsal lateral prefrontal cortex (DLPFC); yellow, anterior cingulate cortex (ACC); violet, median prefrontal cortex (MPFC); cyan, amygdala; orange, insula.

Three main contrasts were specified for subject-level analysis: (1) Fat vs. Control, (2) Thin vs. Control, and (3) Fat vs. Thin. A general linear model was used to generate the statistical parametric maps for the second-level analysis. Main effects were considered significant using a whole-brain false discovery rate (FDR) of *p* < .05 and a minimum cluster size of 10 voxels. Then, eight ROIs were selected based on previous human neuroimaging studies of body processing [8, 9, 19, 20–22, 24–25, 47]. ROI analysis were performed using 10mm spheres generated around a central coordinate taken from previous research. The following Talairach coordinates were selected for each ROI (Fig 1): MPFC (±6, 48, 18); IPL (±51, -30, 40); EBA (±45, -65, 2); FBA (±40, -43, -17); DLPFC (±48, 7, 36); ACC (±11, 23, 23); amygdala (±29, -7, -20) and insula (±29, 19, 7) [8, 19, 48–50].

Using analysis of variance (ANOVA), the beta values of the Fat, Thin and the Control conditions for each ROI were extracted to be incorporated in a general linear model comprising the four cells of the 2 (Groups: O-W vs. A-W group) × 2 (Conditions: Fat-Control vs. Thin-Control) ANOVA. Main effects and interactions between these two variables were computed. The Bonferroni method was used to correct for multiple comparisons [significance level *p <* 0.003 (0.05/16)]. The first hypothesis could be supported if the main effects of Group showed significant differences within ROIs relating to affective components of body image processing. Specifically, we expected that compared to the A-W group, the O-W group would show greater activation in Fat-Control or Thin-Control contrast within ROIs relating to affective components of body image processing. Then, to test the second hypothesis, path analysis based on linear regression were conducted within that/those ROI(s), with BMI as independent variable, beta values of that/those ROI(s) as dependent variable, and body dissatisfaction scores derived from the NPS-F questionnaire as the mediator. Only the ROIs that showed significant Group differences in the corresponding contrasts were used. The third hypothesis would be supported if the main effect of Group or the Group × Condition interaction would have an significant effect on the neural response to fat or thin body images within MPFC.

## Results

### Group characteristics

Compared to the A-W group, the O-W group had a significantly higher score on NPS-F and DEBQ-Restrained eating, as well as a greater level of appearance-related social comparison tendency. Other variables did not differ between the groups (see Table 1).

### Generic group activation maps

**Fig 2:**
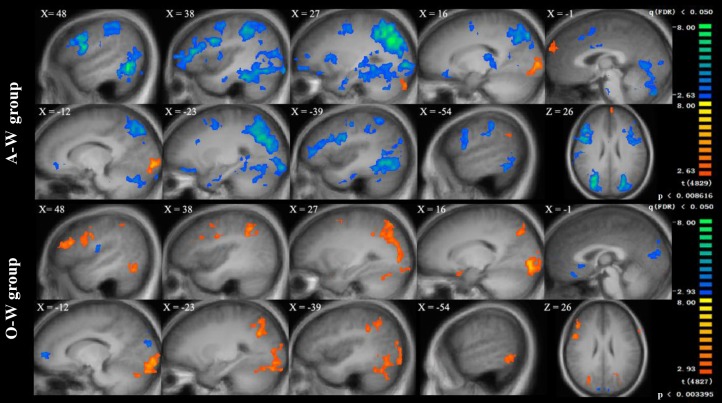
Generic group activation map for fat relative to thin body image self-reflection in 13 Average weight female participants (A-W group) and 13 overweight females participants (O-W group). The depicted activations were associated with the active stimuli at the cluster-wise level of significance at a minimum cluster size of 10 voxels, FDR corrected *p* < 0.05.

For both groups, the Fat-Control and Thin-Control contrasts showed activation of both perceptive and affective brain networks. The perceptive network included bilateral fusiform gyrus (covering FBA), superior and inferior parietal lobule, middle and inferior occipital gyrus (covering EBA) and precuneus. The affective network involved bilateral middle and inferior frontal gyrus, DLPFC, parahippocampal gyrus and amygdala (S1 and S2 Tables). The bilateral MPFC was also activated in Fat-Control and Thin-Control contrasts within the overweight group, whereas MPFC activation was only found in Thin-Control condition within the average weight group. In addition, the groups showed significant differences in brain response in the Fat-Thin condition. Specifically, in this condition the A-W group showed stronger activation in bilateral MPFC, bilateral fusiform gyrus (covering FBA), superior and inferior parietal lobule, middle and inferior occipital gyrus (covering EBA), left precuneus, as well as right DLPFC, bilateral ACC and bilateral amygdala. However, the overweight group showed the reversed pattern, meaning that compared to Thin-body self-reflection, Fat-body self-reflection resulted in stronger activation of bilateral superior parietal lobule, bilateral precuneus, as well as right DLPFC (Fig 2, also see S1 and S2 Tables).

### ROI analysis

**Fig 3:**
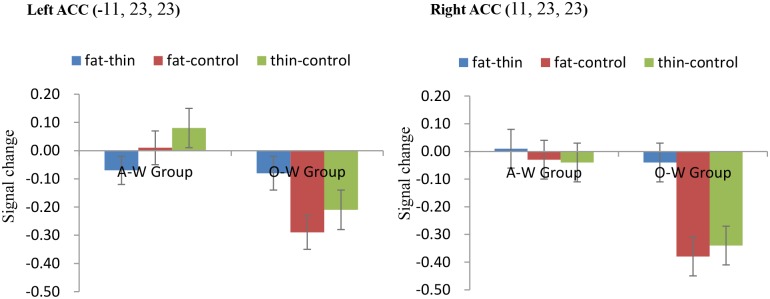
Signal change among Overweight (O-W Group) and Average weight group (A-W Group) across each condition in left and right ACC. Error bars indicate standard error of the mean.

The 2 (Groups: O-W vs. A-W group) × 2 (Conditions: Fat-Control vs. Thin-Control) ANOVA revealed a significant main effect of Group within bilateral ACC [left ACC, *F* (1, 24) = 11.11, *p* < .05; right ACC, *F* (1, 24) = 11.72, *p* < .05, corrected]. A-W group showed a significantly stronger activation in bilateral ACC than the O-W group in both Fat-Control and Thin-Control conditions (Fig 3).

**Fig 4:**
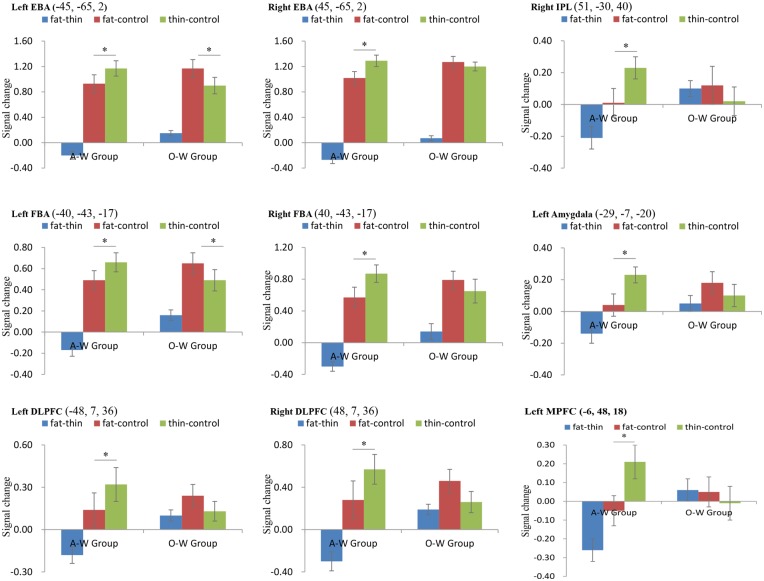
Signal change among Overweight (O-W Group) and Average weight group (A-W Group) across each condition in eight ROIs. Error bars indicate standard error of the mean.

Also, significant Group × Condition interactions were found within bilateral EBA [left EBA, *F* (1, 24) = 34.43, *p* < .05; right EBA, *F* (1, 24) = 19.75, *p* < .05, corrected], bilateral FBA [left FBA, *F* (1, 24) = 128.47, *p* < .05; right FBA, *F* (1, 24) = 12.87, *p* < .05, corrected], right IPL [*F* (1, 24) = 13.05, *p* < .05, corrected], bilateral DLPFC [left DLPFC, *F* (1, 24) = 14.81, *p* < .05; right DLPFC, *F* (1, 24) = 24.79, *p* < .05, corrected], left amygdala [*F* (1, 24) = 11.46, *p* < .05, corrected] and left MPFC [*F* (1, 24) = 14.21, *p* < .05, corrected]. Simple effect analysis within the ROIs mentioned above were conducted. This revealed that within the A-W Group, activation in Thin-Control was stronger than in Fat-Control condition within bilateral EBA, bilateral FBA, right IPL, bilateral DLPFC, left amygdala and left MPFC (all *p*s < 0.05, corrected). Within the O-W Group, Fat-Control resulted in stronger activation than Thin-Control within the left EBA and left FBA (all *p*s < 0.05, corrected, see Fig 4).

### Mediational effect of body dissatisfaction

The path analysis method outlined by Baron and Kenny (1986) [51] was used (for more details, please refer to S1 File). This path analysis was carried out within the ROIs for which a significant main effect or interaction was found.

Based on the results of ROI analysis, activation of bilateral ACC in both Fat-Control and Thin-Control contrasts were introduced as dependent variables. Activation of bilateral EBA, bilateral FBA, bilateral DLPFC, right IPL, left amygdala and left MPFC in Fat-Thin contrast were extracted as dependent variables in path analysis since significant interaction effects were found in these areas. Three steps of linear regressions were conducted to assess whether bodyweight dissatisfaction (NPS-F) functioned as a mediator in the reported group differences.

**Fig 5:**
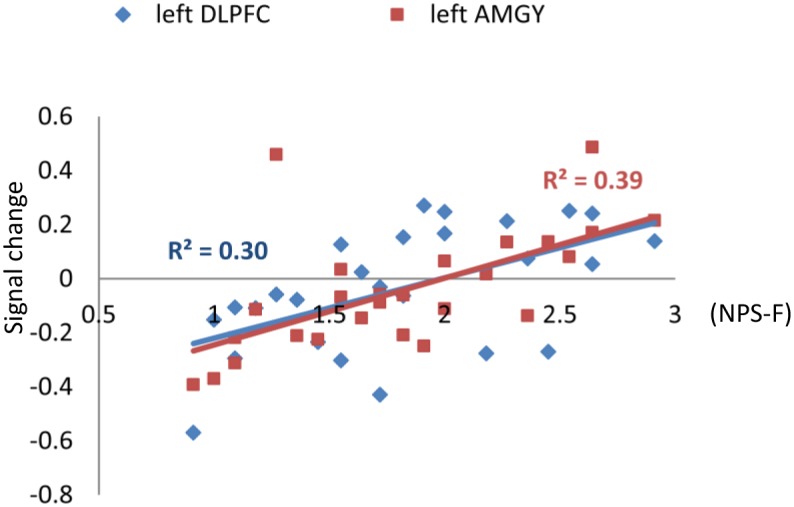
Positive correlations between NPS-F scores and neural activations during fat vs. thin body image self-reflection in left amygdala (x = -29, y = -7, z = -20) and left DLPFC (x = -48, y = 7, z = 36). Significant p value was corrected by the Bonferroni method, that significant differences were determined if the *p* < .05/16 = 0.003.

The analysis of mediational pathways were summarized in Table 2. Path analysis showed that only within the left amygdala, NPS-F completely mediated the group difference in *β* values in the Fat-Thin contrast. Including NPS-F as a mediator made the model explain an additional 13% of variance in signal change. NPS-F also significantly correlated with left DLPFC *β* values in Fat-Thin contrast. However, no mediational effect of NPS-F was observed within this area. No other significant correlations were found. The correlations between NPS-F, left amygdala and left DLPFC *β* values in Fat-Thin contrast are presented in Fig 5.

**Table 2:** Testing mediational effects of bodyweight dissatisfaction within each ROI in Fat-Thin contrast.

Region	Coordinates			IV	DV	*β*	*R*^2^	Δ*R*^2^
	*x*	*y*	*z*					
**Left AMYG (Fat-Thin)**	-29	-7	-20					
Step 1				BMI	AMYG	.52***	.27	
Step 2				NPS-F	AMYG	.63**		
Step 3				BMI+NPS-F	AMYG	(NS) BMI + .63**	.40	.13
**Left DLPFC (Fat-Thin)**	-48	7	36					
Step 1				BMI	DLPFC	.61**	.37	
Step 2				NPS-F	DLPFC	.55**		
Step 3				BMI+NPS-F	DLPFC	.61*** + (NS) NPS-F	.37	0

Note: MPFC = Medial prefrontal cortex; AMYG = Amygdala; DLPFC = Dorsolateral prefrontal cortex. NPS-F = Negative Physical Self Scale-Fatness subscale. IV = Independent variable; DV = Dependent variable; *R*^2^ = Determination coefficient; NS = Not significant.

**p < .01,

***p < .001.

## Discussion

The current *f*MRI study investigated the neural bases of body image processing in overweight young women. The first goal was to investigate whether brain regions that were previously found to be involved in body image processing would show differences in activation between overweight and average weight young women when performing a body image self-reflection task. When such differences would be found, the second goal was to determine whether body dissatisfaction would mediate these group differences in brain response to body image processing.

Consistent with previous findings [8, 10, 12, 17, 18], the used body image self-reflection task successfully activated both perceptive components (IPL, EBA and FBA) and affective components (prefrontal cortex-limbic/paralimbic structures) of body image processing across both groups. These findings suggested that body image processing produced a common pattern of activation in both overweight and average weight women. However, the two groups showed different brain activation patterns in Fat vs. Thin contrast. Specifically, whole brain analysis showed that in A-W group, Thin images resulted in stronger activation than Fat images in a large number of brain regions. These brain regions included a brain network representing perceptive processing of body image (left IPL, bilateral fusiform gyrus covering FBA, left middle and inferior occipital gyrus covering EBA and left precuneus) as well as affective components of body image processing (prefrontal cortex–limbic/paralimbic structures including bilateral DLPFC, bilateral insula and left amygdala). On the other hand, O-W group showed an opposite activation pattern. Among overweight women, Fat images resulted in stronger activation than Thin images in brain regions related to processing perspective components of body image (left FBA, left EBA and bilateral precuneus). Furthermore, ROI analysis confirmed these findings.

A previous study has suggested that EBA and FBA represent different levels of body image processing. The initial processing of whole body images is performed in the EBA, where bodies are processed at the level of separate parts. The next level of processing is performed in the FBA, where the body identity is assigned [18]. The different activation patterns among the two groups in this study might suggest that overweight women showed a stronger visual processing of fat bodies than thin bodies, whereas average weight women showed stronger visual processing of thin body than fat body [52]. These differences in perceptive processing of body image occurred at the initial level, where body parts are processed, as well as at the second level, where whole body identity is processed. The increased perceptual processing may provide the basis for body dissatisfaction or body image disturbance among young females.

Affective processing of body image happens when the visual stream flows from occipital lobe to frontal lobe. Both groups showed significant activation within the affective processing networks by both categories of body pictures. Amygdala and DLPFC were activated in the Fat vs. Control and Thin vs. Control contrasts among both groups. However, the groups showed different activation patterns in the Fat vs. Thin contrast. Specifically, Thin vs. Fat contrast had stronger activation in the left amygdala and bilateral DLPFC in the A-W group, whereas the O-W group showed a trend of the reverse activation pattern in these two areas. Most interestingly, we found a positive correlation between body dissatisfaction level and activation in left amygdala and left DLPFC in Fat vs. Thin contrast within the entire sample. In addition, body dissatisfaction completely mediated the group activation differences in left amygdala activation in Fat vs. Thin condition. When looking at another woman’s body, higher activity of amygdala was suggested to be a neural correlate of negative emotional activation, possibly resulting from unfavorable social comparison processes [34]. Meanwhile, DLPFC activation in body image processing was suggested to reflect the suppression of negative emotions or automatic beliefs about body shape and slimness [34]. The amygdala–frontal circuit was found to be involved in emotion generation and regulation in previous animal and human studies [53–55]. Left amygdala activity was found to co-vary with activity in the DLPFC during reappraisal-based control of negative affect. The current findings in average weight women may suggest a greater negative emotional response to Thin vs. Fat body image self-reflection, which could indicate the possible existence of substantial body dissatisfaction in healthy women [34]. Meanwhile, the overweight women showed a lower ACC activation than the average weight women in both Thin vs. Control and Fat vs. Control condition. In previous studies, ACC activation was found upon exposure to one’s own distorted body image [13, 22] or other women’s overweight image [10]. Reduced ACC activation was also reported among AN patients when they were exposed to body images of slim fashion models [33]. The dorsal part of ACC (BA32) was found to be involved in cognitive conflict monitoring [56, 57]. The reduced ACC activation among the O-W women upon exposure to both fat and thin body images may suggest decreased conflict monitoring during body image self-reflection in overweight young females. The higher activation in amygdala and DLPFC along with a reduced activation in ACC in overweight women may result in their vulnerability to body image distress when confronted with body images in the mass media.

We also found that the two groups differed in activation in MPFC during the body image reflection task. Specifically, bilateral medial frontal regions were activated in both Fat vs. Control and Thin vs. Control conditions within the overweight group, while they were only activated in the Thin vs. Control condition within the average weight group. Since MPFC was consistently found to be involved in self-reflection and perspective taking [9, 10, 21, 23, 58], a more likely explanation is that both groups, when confronted with attractive thin body pictures, appeared to be engaged in self-reflection. However, when confronted with unattractive fat body pictures, only overweight women were engaged in self-reflection or perspective taking, whereas average weight women did not [59, 60]. Activation in MPFC by both fat and thin conditions may have mediated the negative impact of appearance based self-comparison when overweight women were confronted with both types of body shapes.

A limitation of our study is that the current study did not measure state body dissatisfaction during the body image self-reflection task, which limited the interpretation of our neuroimaging results. Second, Asian people’s body image stimuli should be utilized in our future studies to minimize the racial bias. Third, this task was successful in significant self-reflective processing of body image, but the explanation of the current results should be limited in this context. Therefore, future research, using different paradigms that distinguish between the specific components of body image processing, are needed to investigate perceptive and affective components of body image processing among obese or overweight young females and average weight young females.

Our research provides clear evidence that overweight young women process visual stimuli of Fat vs. Thin body images differently from average weight young women during a body image self-reflection task. Overweight females were more likely to engage in self-reflection in the presence of both fat and thin body images, while average weight females seemed less likely to engage in self-reflection when exposed to fat body images. Meanwhile, when comparing Fat vs. Thin image processing, overweight and average weight women showed reversed activation patterns within brain regions related to perspective components of body image processing. Also, we found that body dissatisfaction positively correlated with activation in left amygdala and left DLPFC in the Fat vs. Thin condition. However, overweight females showed decreased emotion and conflict monitoring during Fat vs. Thin body image self-reflection, which was indicated by a lower ACC activation than found in average weight females. Our data are the first to demonstrate a clear group difference in brain activation pattern when overweight and average weight young females are involved in body image self-reflection. These results provide insights for understanding the vulnerability to body image distress among the overweight or obese young females.

## Supporting Information

S1 File Path analysis. (DOCX)

S1 Table Areas with significant activation during the body image self-reflective task among overweight group. (DOCX)

S2 Table Areas with significant activation during the body image self-reflective task among average weight group. (DOCX)
