# Correction: My Body Looks Like That Girl's: Body Mass Index Modulates Brain Activity during Body Image Self-Reflection among Young Women

**DOI:** 10.1371/journal.pone.0172250

**Published:** 2017-02-09

**Authors:** Xiao Gao, Xiao Deng, Xin Wen, Ying She, Petra Corianne Vinke, Hong Chen

The Data Availability statement for this paper is incorrect. The correct statement is: Data are publicly available from the OpenfMRI repository under accession number ds000213 (https://openfmri.org/dataset/ds000213/).
