# Supplementary material for: My Body Looks Like That Girl’s: Body Mass Index Modulates Brain Activity during Body Image Self-Reflection among Young Women
Source: PLoS One. 2016 Oct 20;11(10):e0164450. doi: 10.1371/journal.pone.0164450 (PMC5072594; doi:10.1371/journal.pone.0164450)
Supplement: S1 File — (DOCX) [file pone.0164450.s001.docx]

Path analysis

The path analysis method outlined by Baron and Kenny (1986) was used. For a variable to be a mediator of the relationship between independent variable (IV) and dependent variable (DV), several conditions have to be met. (1) IV must significantly predict DV. (2) Mediator must significantly predict DV. If it does not, then there is no relationship to mediate. (3) IV must significantly predict Mediator. (4) Mediator must significantly predict DV after controlling for IV. If, by adding Mediator to the prediction of DV from IV in a regression model, the effect of IV falls close to zero then full or complete mediation occurred. If the effect of introducing Mediator is reduced by a non-trivial amount but not to zero, partial mediation occurred. If the effect of IV on DV is not reduced upon the addition of Mediator, there is no mediation.

Two mediational pathways were tested, namely (1) BMI—bodyweight dissatisfaction—left amygdala and (2) BMI—bodyweight dissatisfaction—left DLPFC.

Baron, R. M., & Kenny, D. A. (1986). The moderator-mediator variable distinction in social psychological research: conceptual, strategic, and statistical considerations. *J Pers Soc Psychol, 51*(6), 1173-1182.
