# Supplementary material for: My Body Looks Like That Girl’s: Body Mass Index Modulates Brain Activity during Body Image Self-Reflection among Young Women
Source: PLoS One. 2016 Oct 20;11(10):e0164450. doi: 10.1371/journal.pone.0164450 (PMC5072594; doi:10.1371/journal.pone.0164450)
Supplement: S1 Table — (DOCX) [file pone.0164450.s002.docx]

S1 Table. Areas with significant activation during the body image self-reflective task among overweight group

| **Region** | **L/R** | **BA** | **Talairach's coordinates** | | | ***t*** | **cluster size** |
| --- | --- | --- | --- | --- | --- | --- | --- |
|  |  |  | x | y | z |  |  |
| **Overweight Group** |  |  |  |  |  |  |  |
| **Fat > control** |  |  |  |  |  |  |  |
| Middle Frontal Cortex | L | 9, 46 | -44 | 44 | 6 | 4.83 | 2944 |
|  | R | 9, 46 | 47 | 39 | 14 | 8.99 | 4294 |
| Inferior Frontal Gyrus | L | 45 | -47 | 24 | 17 | 5.85 | 1813 |
|  | R | 45 | 41 | 21 | 14 | 7.10 | 957 |
| Lateral OFC | L | 47 | -30 | -25 | 11 | 6.27 | 1253 |
|  | R | 47 | 35 | 30 | -6 | 5.05 | 1507 |
| DLPFC | L | 9 | -48 | 7 | 36 | 7.01 | 1913 |
|  | R | 9 | 49 | 4 | 31 | 11.13 | 2356 |
| Medial Frontal Cortex | L | 6 | -5 | 6 | 50 | 8.14 | 3815 |
|  | R | 6 | 6 | -1 | 59 | 5.92 | 2541 |
| Precentral Gyrus | L | 6 | -36 | 11 | 36 | 8.77 | 1562 |
|  | R | 6 | 38 | 1 | 31 | 6.52 | 1621 |
| Parahippocampal Gyrus | L | 46 | -36 | -30 | -16 | 7.93 | 1015 |
|  | R | 46 | 33 | -30 | -20 | 8.67 | 1374 |
| Amygdala | L | — | -31 | -4 | -22 | 5.15 | 1086 |
|  | R | — | 30 | -2 | -22 | 6.51 | 1526 |
| Caudate body | L | — | -15 | 4 | 16 | 5.23 | 624 |
| Putamen | L | — | -20 | -4 | 10 | 6.34 | 935 |
| Lateral Globus Pallidus | L | — | -19 | -3 | 10 | 6.28 | 595 |
|  | R | — | 18 | -3 | 9 | 5.45 | 426 |
| Thalamus | L | — | -18 | -30 | 2 | 5.15 | 302 |
|  | R | — | 17 | -28 | 3 | 7.67 | 1655 |
| Superior Parietal Lobule | L | 7 | -32 | -56 | 50 | 7.01 | 893 |
|  | R | 7 | 31 | -56 | 47 | 6.15 | 1054 |
| Precuneus | L | 7 | -11 | -72 | 45 | 6.47 | 656 |
|  | R | 7 | 6 | -74 | 44 | 9.33 | 925 |
| Inferior Parietal Lobule | L | 7, 40 | -33 | -57 | 48 | 8.04 | 2496 |
|  | R | 7, 40 | 33 | -56 | 45 | 8.13 | 2656 |
| Cuneus | L | 17 | -21 | -88 | 8 | 12.79 | 843 |
|  | R | 17 | 21 | -88 | 8 | 10.67 | 914 |
| Middle Temporal Gyrus | L | 39 | -41 | -56 | 3 | 9.24 | 502 |
|  | R | 39 | 41 | -56 | 6 | 10.35 | 632 |
| Fusiform Gyrus | L | 20, 37 | -40 | -45 | -17 | 8.42 | 2175 |
|  | R | 20, 37 | 35 | -51 | -14 | 9.64 | 2667 |
| Middle Occipital Gyrus | L | 19 | -33 | -88 | 8 | 13.03 | 769 |
|  | R | 19 | 33 | -85 | 13 | 19.28 | 944 |
| Inferior Occipital Gyrus | L | 18 | -32 | -88 | -5 | 13.67 | 748 |
|  | R | 18 | 30 | -88 | -8 | 11.42 | 869 |
| Cerebellum | L | — | -6 | -66 | -24 | 6.91 | 2165 |
|  | R | — | 4 | -70 | -25 | 5.22 | 1553 |
| **Thin > control** |  |  |  |  |  |  |  |
| Superior Frontal Cortex | L | 9 | -11 | 54 | 31 | 4.36 | 428 |
| Middle Frontal Cortex | L | 6 | -48 | 1 | 42 | 8.37 | 2045 |
|  | R | 6 | 46 | 1 | 34 | 10.31 | 3124 |
| Middle Frontal Cortex | L | 46 | -37 | 29 | 21 | 4.72 | 420 |
|  | R | 46 | 44 | 29 | 19 | 6.14 | 838 |
| Inferior Frontal Cortex | L | 45 | -50 | 19 | 14 | 4.59 | 540 |
|  | R | 45 | 41 | 23 | 14 | 4.31 | 327 |
| Lateral OFC | L | 47 | -40 | 39 | -2 | 6.13 | 1324 |
|  | R | 47 | 34 | 32 | -2 | 4.30 | 336 |
| DLPFC | L | 9 | -48 | 8 | 30 | 6.21 | 822 |
|  | R | 9 | 41 | 21 | 27 | 10.47 | 1356 |
| Medial Frontal Cortex | L | 6 | -6 | 10 | 55 | 5.60 | 2534 |
|  | R | 6 | 4 | -1 | 57 | 4.84 | 383 |
| Precentral Gyrus | L | 6 | -36 | -2 | 39 | 8.78 | 961 |
|  | R | 6 | 46 | -7 | 38 | 8.75 | 2013 |
| Amygdala | L | — | -34 | -2 | -21 | 6.02 | 640 |
|  | R | — | 30 | -2 | -20 | 5.11 | 316 |
| Thalamus | R | — | -17 | -27 | 6 | 8.25 | 628 |
| Superior Parietal Lobule | L | 7 | -32 | -56 | 50 | 6.98 | 801 |
|  | R | 7 | 24 | -56 | 55 | 7.6.24 | 994 |
| Precuneus | L | 7 | -24 | -56 | 21 | 7.02 | 612 |
|  | R | 7 | 14 | -61 | 52 | 8.23 | 803 |
| Inferior Parietal Lobule | L | 7, 40 | -33 | -56 | 52 | 7.34 | 2036 |
|  | R | 7, 40 | 30 | -56 | 49 | 8.34 | 2155 |
| Cuneus | L | 17 | -20 | -88 | 10 | 10.38 | 654 |
|  | R | 17 | 19 | -87 | 8 | 11.44 | 532 |
| Middle Temporal Gyrus | L | 39 | -42 | -62 | 14 | 8.21 | 569 |
|  | R | 39 | 46 | -62 | 17 | 9.34 | 521 |
| Fusiform Gyrus | L | 20 | -40 | -40 | -13 | 8.42 | 2199 |
|  | R | 20 | 38 | -37 | -13 | 8.50 | 2635 |
| Middle Occipital Gyrus | L | 18 | -32 | -87 | -1 | 12.39 | 701 |
|  | R | 18 | 31 | -81 | 3 | 15.54 | 869 |
| Inferior Occipital Gyrus | L | 18 | -32 | -88 | -5 | 11.24 | 523 |
|  | R | 18 | 30 | -87 | -5 | 14.09 | 588 |
| Cerebellum | L | — | -8 | -69 | -28 | 6.07 | 442 |
| **Fat > Thin** |  |  |  |  |  |  |  |
| Middle Frontal Cortex | R | 46 | 47 | 34 | 22 | 5.56 | 792 |
| Lateral OFC | R | 47 | 17 | 10 | -15 | 5.44 | 275 |
| Precentral Gyrus | R | 6 | 37 | -3 | 33 | 6.04 | 857 |
| Superior Parietal Lobule | L | 7 | -26 | -64 | 48 | 5.91 | 1531 |
|  | R | 7 | 30 | -64 | 48 | 4.97 | 1836 |
| Insula | R | 13 | 33 | 17 | 3 | 4.21 | 287 |
| Precuneus | L | 7 | -22 | -65 | 37 | 5.85 | 1264 |
|  | R | 7 | 25 | -61 | 32 | 5.80 | 1915 |
| Middle Occipital Gyrus | L | 19 | -33 | -79 | 16 | 4.98 | 487 |
|  | R | 19 | 28 | -79 | 11 | 5.44 | 913 |
| Lingual Gyrus | L | 18 | -16 | -83 | -13 | 7.63 | 1775 |
|  | R | 18 | 14 | -83 | -5 | 6.41 | 1567 |
| Cerebellum | L | — | -37 | -61 | -26 | 6.14 | 529 |
| **Thin > Fat** |  |  |  |  |  |  |  |
| Anterior Cingulate Cortex | L | 24 | -5 | 23 | -4 | 5.68 | 565 |
| Anterior Cingulate Cortex | L | 25 | 0 | 19 | 0 | 5.51 | 270 |
| Cuneus | L | 18 | 9 | -78 | 16 | 5.01 | 966 |
|  | R | 17 | -4 | -78 | 13 | 4.21 | 485 |
| Superior Temporal Gyrus | R | 22, | 53 | -51 | 16 | 4.31 | 577 |

Activation threshold *p* < .05, FDR corrected, at a minimum cluster size of 10 voxels.
