# Supplementary material for: My Body Looks Like That Girl’s: Body Mass Index Modulates Brain Activity during Body Image Self-Reflection among Young Women
Source: PLoS One. 2016 Oct 20;11(10):e0164450. doi: 10.1371/journal.pone.0164450 (PMC5072594; doi:10.1371/journal.pone.0164450)
Supplement: S2 Table — (DOCX) [file pone.0164450.s003.docx]

S2 Table. Areas with significant activation during the body image self-reflective task among average weight group

| **Region** | **L/R** | **BA** | **Talairach's coordinates** | | | ***t*** | **cluster size** |
| --- | --- | --- | --- | --- | --- | --- | --- |
|  |  |  | x | y | z |  |  |
| **Fat > Control** |  |  |  |  |  |  |  |
| Superior Frontal Gyrus | L | 6 | -3 | 5 | 61 | 9.27 | 3754 |
| Middle Frontal Gyrus | R | 46 | 39 | 29 | 13 | 5.72 | 1072 |
| Middle Frontal Gyrus | L | 6 | -33 | -1 | 46 | 8.89 | 1117 |
|  | R | 6 | 33 | -10 | 46 | 7.54 | 2056 |
| Inferior Frontal Gyrus | L | 9 | -33 | 11 | 24 | 5.64 | 568 |
| DLPFC | R | 9 | 48 | 8 | 31 | 10.77 | 1698 |
| OFC | R | 11 | 3 | 29 | -11 | 4.20 | 484 |
| Parahippocampal Gyrus | L | 28 | -15 | -4 | -11 | 5.79 | 901 |
| Caudate Body | L | — | -17 | 11 | 9 | 5.95 | 302 |
| Amygdala | R | — | 33 | -4 | -25 | 5.78 | 399 |
| Medial Globus Pallidus | L | — | -18 | -7 | 1 | 6.15 | 751 |
|  | R | — | 15 | -7 | -8 | 5.79 | 510 |
| Posterior Cingulate | L | 31 | -6 | -52 | 22 | 4.02 | 458 |
| Thalamus | L | — | -18 | -28 | 4 | 6.14 | 613 |
|  | R | — | 15 | -28 | 1 | 4.71 | 329 |
| Inferior Temporal Gyrus | L | 21 | -57 | -7 | -11 | 5.54 | 823 |
| Superior Parietal Lobule | L | 7 | -21 | -67 | 46 | 13.94 | 2468 |
| Inferior Parietal Lobule | L | 7 | -33 | -58 | 47 | 8.32 | 1658 |
| Precuneus | L | 7, 19 | -24 | -75 | 31 | 9.42 | 1983 |
|  | R | 7, 19 | 15 | -61 | 47 | 9.22 | 1816 |
| Fusiform Gyrus | L | 20, 37 | -40 | -46 | -18 | 8.22 | 1965 |
|  | R | 20, 37 | 33 | -33 | -18 | 10.67 | 2354 |
| Superior Temporal Gyrus | L | 13 | -43 | -48 | 26 | 5.01 | 601 |
| Middle Temporal Gyru | L | 37 | -44 | -63 | 11 | 11.59 | 1184 |
|  | R | 37 | 48 | 63 | 8 | 12.65 | 1323 |
| Middle Occipital Gyrus | L | 37 | -39 | -63 | 6 | 11.16 | 914 |
|  | R | 37 | 39 | -63 | 4 | 11.52 | 966 |
| Inferior Occipital Gyrus | L | 19 | -39 | -73 | -3 | 18.21 | 601 |
|  | R | 19 | 39 | -73 | -5 | 27.71 | 567 |
| Cerebellum | R | — | 27 | -70 | -29 | 5.64 | 710 |
| **Thin > Control** |  |  |  |  |  |  |  |
| Superior Frontal Gyrus | L | 6,8,9,10 | 0 | 9 | 49 | 7.92 | 2865 |
|  | R | 6 | 4 | 2 | 67 | 7.29 | 652 |
| Medial Frontal Gyrus | L | 6,8,10,11,25 | 0 | 62 | 4 | 6.29 | 1584 |
|  | R | 10,11,32 | 4 | 53 | -5 | 7.61 | 1293 |
| DLPFC | L | 9 | -50 | 6 | 32 | 8.02 | 2001 |
|  | R | 9 | 53 | 6 | 32 | 7.33 | 2455 |
|  | R | 46 | 40 | 29 | 19 | 8.24 | 2154 |
| Postcentral Gyrus | L | 7 | -13 | -53 | 63 | 7.43 | 583 |
| Anterior Cingultate Cortex | L | 25,32,33 | 0 | 9 | -8 | 5.20 | 1018 |
| Posterior Cingulate Cortex | L | 23 | -2 | -47 | 24 | 4.02 | 235 |
| Cingulate Gyrus | L | 24,31,32 | -2 | 20 | 40 | 7.13 | 1698 |
|  | R | 24,32 | 6 | 8 | 26 | 5.44 | 455 |
|  | R | 7 | 4 | -73 | 43 | 7.25 | 347 |
| Caudate | L | — | -17 | 7 | 16 | 5.29 | 1121 |
|  | R | — | 17 | 2 | 16 | 4.80 | 722 |
| Lentiform Nucleus | L | — | -10 | -2 | 3 | 6.29 | 468 |
| Amygdala | L | — | -31 | -2 | -25 | 5.74 | 1012 |
|  | R | — | 28 | -2 | -24 | 6.65 | 895 |
| Thalamus | L | — | -11 | -17 | 11 | 5.61 | 1014 |
|  | R | — | 13 | -17 | 10 | 5.73 | 925 |
| Superior Parietal Lobule | L | 7,9 | -2 | 53 | 27 | 5.19 | 424 |
|  | R | 7 | 4 | -66 | 54 | 6.48 | 393 |
| Precuneus | L | 7,23,31 | 0 | -59 | 19 | 8.57 | 1339 |
| Cuneus | L | 17,18 | -16 | -82 | 9 | 9.05 | 859 |
| Lingual Gyrus | L | 17,18,19 | -21 | -85 | 6 | 15.18 | 1994 |
| **Fat > Thin** |  |  |  |  |  |  |  |
| Superior Frontal Gyrus | L | 9 | -3 | 53 | 28 | 4.3 | 401 |
| Lingual Gyrus | L | 17 | -15 | -91 | -2 | 7.09 | 2642 |
|  | R | 17 | 12 | -91 | 1 | 7.25 | 1510 |
| Cerebellum | R | — | 24 | -79 | -20 | 4.79 | 651 |
| **Thin > Fat** |  |  |  |  |  |  |  |
| Superior Frontal Gyrus | R | 6,10 | 2 | 9 | 49 | 4.84 | 447 |
| Middle Frontal Gyrus | L | 6,9,11 | -22 | -1 | 44 | 5.69 | 1233 |
| Inferior Frontal Gyrus | L | 47 | -18 | 30 | -15 | 4.79 | 144 |
| Medial Frontal Cortex | L | 8 | 0 | 24 | 43 | 5.21 | 651 |
|  | R | 8,10 | 2 | 22 | 43 | 4.74 | 681 |
| Insula | L | 13 | -24 | 29 | 8 | 5.03 | 321 |
|  | R | 13 | 30 | 24 | 6 | 4.96 | 389 |
| Putamen | R | — | 26 | 12 | 6 | 5.92 | 443 |
| Uncus | L | 20 | -32 | -9 | -25 | 6.14 | 618 |
| Amygdala | L | — | -34 | -7 | -21 | 5.65 | 529 |
|  | R | — | 30 | -2 | -25 | 5.13 | 753 |
| Cingulate Gyrus | L | 24 | 0 | 9 | 31 | 3.94 | 600 |
|  | R | 24 | 2 | 2 | 33 | 5.41 | 379 |
| Anterior Cingulate Cortex | L | 32 | -4 | 36 | -6 | 5.06 | 253 |
|  | R | 32 | 17 | 39 | -8 | 5.45 | 345 |
| Posterior Cingulate Cortex | L | 30 | 0 | -61 | 9 | 5.00 | 526 |
| Thalamus | R | — | 7 | -15 | 15 | 5.48 | 539 |
| Hippocampus Gyrus | L | 36 | -30 | -9 | -19 | 4.05 | 353 |
| Parahippocampal | L | 36 | -30 | -32 | -10 | 6.22 | 546 |
| Parahippocampal Gyrus | L | 30 | -18 | -40 | 5 | 5.58 | 337 |
| Superior Temporal Gyrus | L | 38 | -22 | 8 | -31 | 4.44 | 173 |
| Fusiform Gyrus | L | 18,19,37 | -30 | -41 | -12 | 7.69 | 1062 |
|  | R | 18,19,37 | 33 | -33 | -16 | 8.21 | 1488 |
| Superior Parietal Lobule | L | 7 | -17 | -56 | 61 | 5.72 | 565 |
|  | R | 7 | 9 | -64 | 56 | 5.00 | 680 |
| Inferior Pareital Lobule | L | 40 | -30 | -40 | 39 | 6.70 | 826 |
| Precuneus | L | 7,31 | -10 | -59 | 45 | 7.05 | 6126 |
| Lingual Gyrus | L | 18 | 0 | -74 | 5 | 4.12 | 134 |
|  | R | 18 | 2 | -80 | 6 | 4.09 | 256 |
| Superior Occipital Gyrus | L | 19 | -30 | -86 | 13 | 5.19 | 661 |
| Inferior Occipital Gyrus | L | 18 | -30 | -86 | -9 | 6.49 | 706 |
| Cerebullum | L | — | -10 | -59 | -23 | 5.52 | 1439 |
|  | R | — | 0 | -43 | -27 | 5.00 | 1373 |

Activation threshold *p* < .05, FDR corrected, at a minimum cluster size of 10 voxels.
